# Contribution to Tumor Angiogenesis From Innate Immune Cells Within the Tumor Microenvironment: Implications for Immunotherapy

**DOI:** 10.3389/fimmu.2018.00527

**Published:** 2018-04-05

**Authors:** Adriana Albini, Antonino Bruno, Douglas M. Noonan, Lorenzo Mortara

**Affiliations:** ^1^Scientific and Technology Pole, IRCCS MultiMedica, Milano, Italy; ^2^Department of Medicine and Surgery, University Milano-Bicocca, Monza, Italy; ^3^Department of Biotechnology and Life Sciences, University of Insubria, Varese, Italy

**Keywords:** angiogenesis, chemoprevention, tumor microenvironment, immune cells, immunotherapy

## Abstract

The critical role of angiogenesis in promoting tumor growth and metastasis is strongly established. However, tumors show considerable variation in angiogenic characteristics and in their sensitivity to antiangiogenic therapy. Tumor angiogenesis involves not only cancer cells but also various tumor-associated leukocytes (TALs) and stromal cells. TALs produce chemokines, cytokines, proteases, structural proteins, and microvescicles. Vascular endothelial growth factor (VEGF) and inflammatory chemokines are not only major proangiogenic factors but are also immune modulators, which increase angiogenesis and lead to immune suppression. In our review, we discuss the regulation of angiogenesis by innate immune cells in the tumor microenvironment, specific features, and roles of major players: macrophages, neutrophils, myeloid-derived suppressor and dendritic cells, mast cells, γδT cells, innate lymphoid cells, and natural killer cells. Anti-VEGF or anti-inflammatory drugs could balance an immunosuppressive microenvironment to an immune permissive one. Anti-VEGF as well as anti-inflammatory drugs could therefore represent partners for combinations with immune checkpoint inhibitors, enhancing the effects of immune therapy.

## Introduction

The “gradient” of phenotype, genetic, and epigenetic features of transformed cells inside the tumor gives rise to the most known and studied tumor heterogeneity, the “intrinsic” one. However, increasing attention is devoted to “extrinsic” heterogeneity, i.e., all those cellular and molecular “players” that include the non-cancerous hosting environment. Cancers develop in complex tissue environments, both in the primary and in the target organs of metastasis. A “hostile” setting is elicited, such as low oxygen, acidity, and altered metabolic conditions. Cancer cells adapt more rapidly than healthy ones to the adverse conditions that paradoxically sustain growth, invasion, and metastasis. In such an “infernal” environment, interactions between tumor cells and the associated stroma represent a dangerous relationship that reciprocally influences disease initiation, progression and, in the end, determines patient prognosis (1).

**Figure 1:**
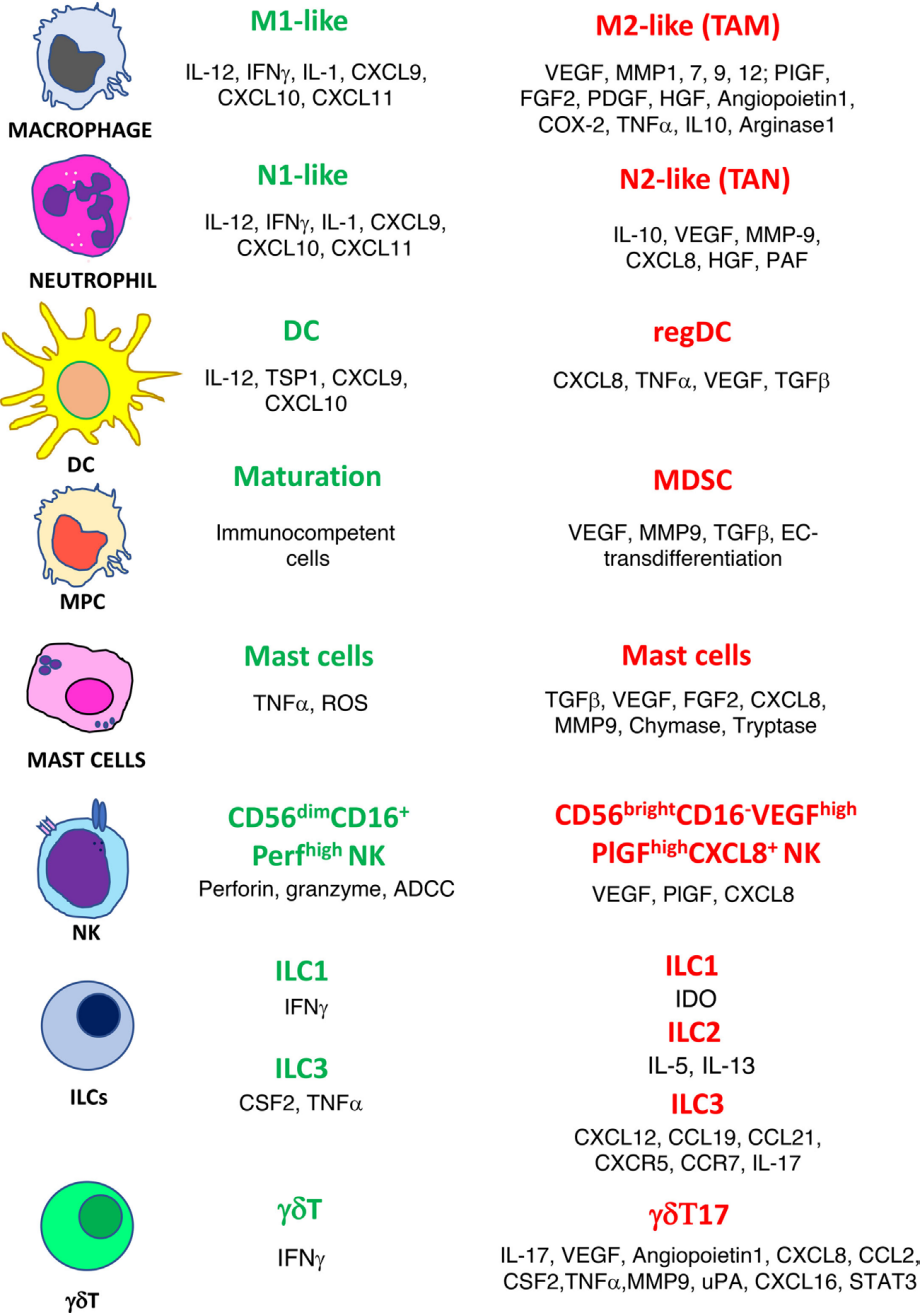
Phenotype switch of innate immune cells in cancer. Antitumor/antiangiogenic (green text) and protumor/proangiogenic (red text) features are listed for macrophages, neutrophils, dendritic cells (DC), myeloid precursor cells (MPC), mast cells, natural killer cells (NK), innate lymphoid cells (ILCs), and γδT cells.

The confirmed theory that the presence of inflammatory cells plays a crucial role within the tumor microenvironment (TME) is a very old one (2). “Evading immune destruction” and “tumor-promoting inflammation” are recognized host-dependent tumor hallmarks as defined by Hanahan and Weinberg (3). Among the tumor-friendly phenomena generated through the activity of the inflammatory cells in the microenvironment, there is the orchestration of angiogenesis, a biological phenomenon necessary to bring oxygen, nutrition to the tumors, and last but not least, to transport the cancer cell to metastatic sites (4–7). Innate immune cells, as a consequence of their plasticity, have been reported to acquire an altered phenotype that can be proangiogenic. For many immune cells, both from innate and adaptive immunity, the release of proangiogenic cytokines is accompanied by a switch to a tolerogenic/immunosuppressive behavior (4, 7–9). In this review, we choose to describe the role in angiogenesis of selected major classes of inflammatory cells: macrophages, neutrophils, myeloid-derived suppressor cells (MDSCs), dendritic cells (DCs), mast cells (MCs), gammadelta (γδ)T type 17 cells (γδT17), innate lymphoid cells (ILCs), and natural killer (NK) cells (Figure 1). We also sustain the rationale behind using antiangiogenic drugs before the onset of immunotherapy and we propose as an innovative, low-cost strategy the use of “repurposed” anti-inflammatory/chemopreventive drugs to assist immunotherapies.

## Macrophages

**Figure 2:**
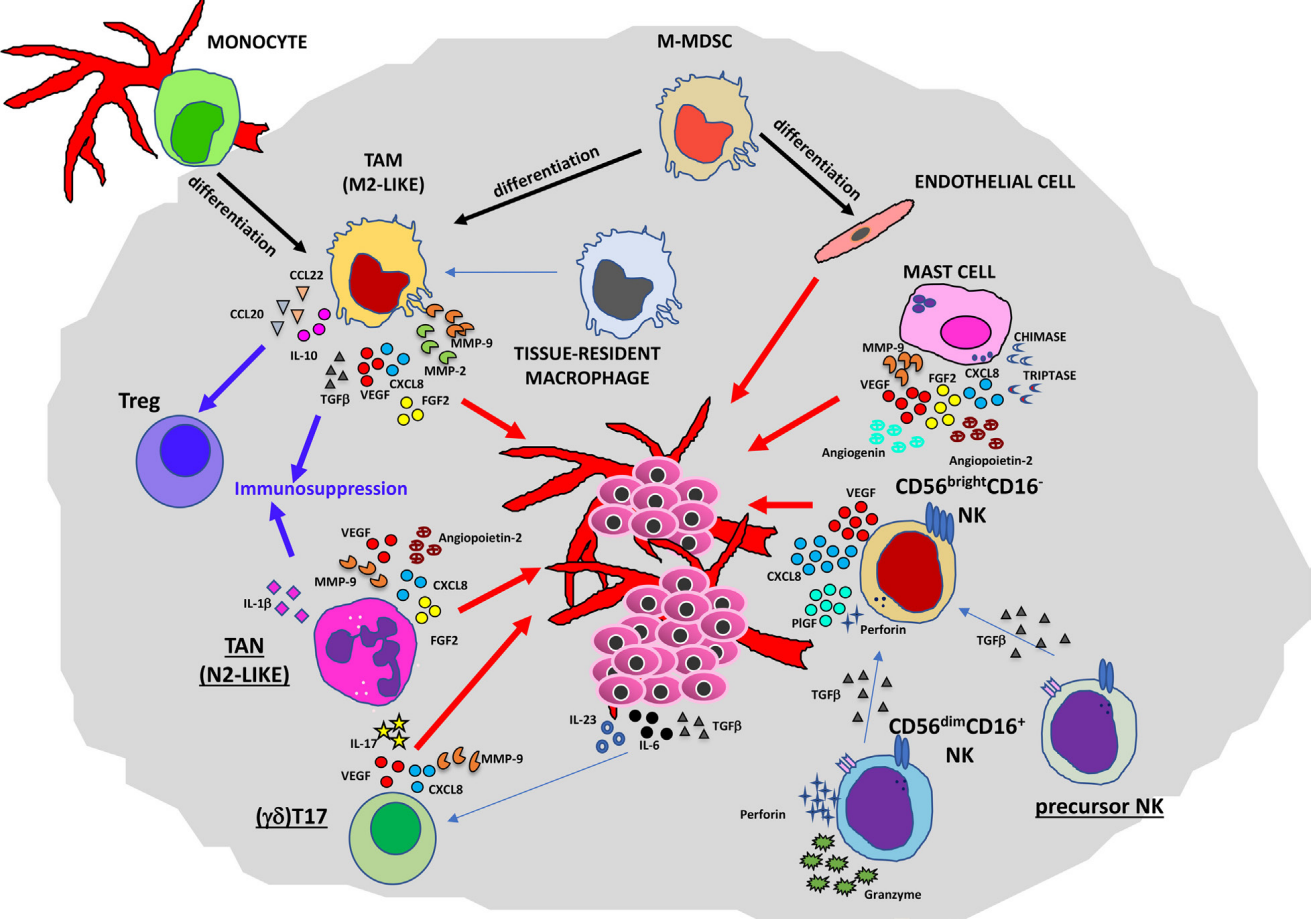
Contribution of innate immunity to tumor angiogenesis. Soluble mediators (chemokines, cytokines, and enzymes) within the tumor microenvironment act directly or indirectly as proangiogenic factors produced by macrophages [M2-like tumor-associated macrophages (TAMs)], neutrophils [tumor-associated neutrophil (TAN), N2-like], myeloid-derived-suppressor cells (MDSCs), mast cells, and natural killer (NK) cells.

Macrophages constitute professional phagocytes of the innate immune cell compartment with different specialized functions, depending on the type of danger signals and endogenous molecules to which they are exposed (10). They act as sentinels in all tissues of the body against invading pathogens, are able to trigger an inflammatory response, and collaborate with other immune cells to activate adaptive T lymphocyte responses through antigen processing and presentation. These activities are related to a classical activation state, which is type 1 T helper (TH1) cell associated and INFγ and/or LPS-dependent, and is referred to as M1. This condition is favorable to immune response. Macrophages can be alternatively activated by IL-4 and/or IL-13 signals from TH2 cells, eosinophils, and/or basophils in the surrounding microenvironment. This polarization is involved in parasite control and wound healing and is termed M2 (11). M2 macrophages are associated with chemical and physical tissue damage in which they mediate tissue homeostasis and repair *via* remodeling and angiogenesis, in a spectrum of differentiation states. *In vivo*, the plasticity and diversity of macrophages are responsible of a spectrum of different activation states strictly depending on an array of concordant but also discordant stimuli, such as hypoxia, chemokines, colony-stimulating factor 1 (CSF1), TGFβ, adenosine, and prostaglandin E2 (PGE2), that do not fit with the M1/M2 classification (12). For these reasons, M1-like is the preferred term used in this review and indicate a polarization state of macrophages that are able to orchestrate cytotoxic antipathogen and antitumor responses, whereas M2-like are cells that have the common functional feature of favoring tumor cell fitness, new blood vessel formation, as well as suppressive activities toward adaptive immune cells (13, 14). Tumor-associated macrophages (TAMs), which share many features with M2-like macrophages (Figure 2), represent the major cell population of tumor-infiltrating leukocytes (15). TAMs also show consistent differences between diverse types of cancers (16, 17). Elevated TAM infiltration has been correlated with poor clinical outcome in many types of cancers, such as ovarian, breast, prostate, cervical, and thyroid cancers, Hodgkin’s lymphoma, cutaneous melanoma, lung, and hepatocellular carcinomas (14, 18–22). Conversely, other reports on colorectal, prostatic, and lung cancers have detected a positive role of infiltrating macrophages favoring increased patient survival (23–25). During cancer development, macrophages are recruited in the tumor stroma by several inflammatory mediators, such as chemokines: CCL2 (also known as MCP-1), CCL5, CXCL12 (also known as SDF-1), cytokines: vascular endothelial growth factor (VEGF), CSF1, and activated complement elements. Blood monocytes, blood monocytic MDSCs, cells, or tissue-resident macrophages (26–28) are subverted in their phenotype and functions to differentiate into TAMs (14). However, TAMs are not fixed in an irreversible phenotype, they maintain their plasticity and eventually could be targeted by specific therapeutic approaches to re-educate them to M1-like antitumor functions (29). Accumulating evidence have shown that TAMs can act as key cellular mediators, interconnecting chronic inflammation with cancer development and progression (3, 30).

Several lines of research have pointed out the role of TAMs in the regulation of tumor cell invasion, angiogenesis, lymphangiogenesis, and metastasis (11, 19). In primary tumors (Figure 2), they can promote angiogenesis (the “angiogenic switch”) triggering the activation and the recruitment of endothelial cells (ECs), essentially by producing multiple proangiogenic factors, including VEGFA, epidermal growth factor (EGF), basic fibroblast growth factor 2 (FGF2), chemokines CXCL8 (also known as IL-8), CXCL12, TNFα, semaphorin 4D, adrenomedullin, and thymidine phosphorylase (31–34). These factors produced by TAMs are responsible for the proliferation of ECs and the induction of sprouting, tube formation, and maturation of new vessels. Macrophages have been shown to play a critical role in tumor lymphangiogenesis by producing VEGFC and VEGFD (35–37). TAM activities can also have an impact on degradation and remodeling of the extracellular matrix (ECM), through the production of different classes of enzymes and proteases, such as matrix metalloproteinases (MMPs in particular MMP2 and MMP9), plasmin, urokinase plasminogen activator (uPA), and cathepsins, thereby influencing tumor invasion and the metastatic process (38–40).

Tumor-associated macrophages are also associated with resistance to different chemotherapeutic agents, involving the activation of distinct molecular pathways. In breast cancers, TAMs are able to inhibit apoptosis of cancer cells upon paclitaxel treatment *via* induction of IL-10/signal transducer and activator of transcription (STAT)3/Bcl-2 signaling (41). In patients with non-small cell lung cancer, TAMs or M2-like TAMs dampen the responsiveness to targeted therapy with EGF receptor–tyrosine kinase inhibitors (42, 43).

A highly proangiogenic M2-like TAM subset is represented by angiopoietin responsive Tie2^+^ perivascular macrophages (35–37), which are able to induce chemotherapeutic drug resistance, favoring decreasing cancer cell responsiveness to radiotherapy (44). Specific inhibition of the angiopoietin/Tie2 axis can act in synergy with antiangiogenic treatments (45). Apart from their proangiogenic features, TAMs also play a crucial role in promoting an immunosuppressive milieu helping different tumors to escape immunosurveillance (46). Their contribution to tumor progression act also through crosstalk with other leukocytes and inflammatory and stromal cells (7, 47) within the TME. In the establishment of the immunosuppressive milieu, TAMs can directly recruit T regulatory (Treg) cells, by producing CCL20 (48) and CCL22 chemokines (49) and can activate them by secreting IL-10 and TGFβ (26). TAMs also represent an important factor for the establishment of the premetastatic niche (50, 51).

Different TAM-targeted therapeutic strategies have been developed with the aim to inhibit macrophage recruitment, to induce cell death, and to re-educate killer functions. These innovative therapeutic approaches could behave as a complement strategy in combination with antiangiogenic, cytoreductive, and/or immune checkpoint inhibitor treatments, and preclinical and clinical trial results are promising (14, 30, 52). CCL2-specific inhibition by antibodies has proven efficacious in mouse models of prostate, breast, lung, and melanoma, and this approach was synergistic with chemotherapy (53, 54). Different antibodies targeting CCL2 have entered phase I and II clinical trials (55). A CCR5 antagonist has been approved for the treatment of patients with liver metastases from advanced colorectal cancers and experimental data indicate that CCL5/CCR5 axis targeting could be suitable for clinical responses (56). Diverse compounds and antibody inhibitors that have been developed to inhibit the CSF1–CSF1R axis, could target TAM, and were evaluated in mouse models and in patients with different types of cancer (57). In diffuse-type tenosynovial giant-cell tumor showing overexpression of CSF1R, after treatment with CSF1R-blocking agents, patients experienced relevant clinical regressions (57, 58). In preclinical glioblastoma multiforme model, CSF1R blockade did not affect the TAM numbers but the M2-like TAM polarization markers were lowered, thus was associated with improvement of survival (59). Bisphosphonates, that are used to treat osteoporosis and to prevent bone metastases-related complications, can also be used to target macrophages inside the tumor (60). Moreover, bisphosphonates in combination with chemotherapy or hormonal therapy have been shown clinical synergistic effects, in different types of cancer patients, in particular for patients with breast cancer (61). In a murine model of pancreatic ductal adenocarcinoma (PDAC), the anti-CD40- and gemcitabine-treated mice induced re-education of M2-like TAM toward an M1-like macrophage and elicit effective antitumor responses (62). This lead to a phase I clinical trial in PDAC patients, the combination was well tolerated and provided some antitumor efficacy (63). A recently identified potent compound that targets TAMs is trabectedin, a synthetic form of a molecule isolated from the marine tunicate *Ecteinascidia turbinata*, which has found application in the treatment of soft tissue sarcomas and ovarian cancer patients. Trabectedin induces selective TRAIL-dependent apoptosis of monocytes, macrophages, and the monocytic component of MDSCs in blood, spleen, and tumors with the reduction of TAM numbers and angiogenesis (64, 65).

## Neutrophils

Neutrophils are the most abundant innate immune cells in the peripheral blood, they act as a first line of defense against invading pathogens and are crucial effectors in the acute phase of inflammation. Neutrophils are recruited in the damaged area by chemokines, in particular CXCL8, and the cognate receptors CXCR1 and CXCR2 (66). These leukocytes exert important functions such as phagocytosis, production and release of antimicrobial ROS, peptides, enzymes, and neutrophil extracellular traps (NET). Neutrophils can release a substantial quantity of different reactive soluble factors, including cytokines and chemokines (67), and are able to recruit and activate other immune cells, playing an important role in the regulation of chronic inflammation, tumor angiogenesis, and progression. Inflammatory CD66b^+^ neutrophils can be found in high numbers in either blood or TME of different cancers and correlated with poor clinical outcome (68–74).

Neutrophils produce either proangiogenic or antiangiogenic factors (75–79), and in some cases, such as in the early phases of lung cancers, they can exert important T cell stimulatory, antitumor functions (80). Although they are characterized by a terminally differentiated phenotype and a short half-life, these cells are endowed with a certain kind of plasticity and in murine tumor models they are able to differentiate in two distinct subsets: neutrophils type 1 (N1) with antimicrobial functions, and tumor-associated neutrophils (TANs or N2) endowed with protumor and proangiogenic features (Figure 2) in response to TGFβ (81, 82). In response to IFNβ, TAN/N2 neutrophils can be converted to N1 type in both mouse lung cancers and human melanomas (83, 84).

Accumulating evidence has indicated TANs as key players involved in tumor angiogenesis and metastatic process in both mice and humans (Figure 2). The complex role of TANs in tumor angiogenesis and metastasis resides mainly in the capacity of these cells to secrete an array of diverse immunosuppressive or proangiogeneic molecules such as IL-1β, VEGF, FGF2, TGFα, hepatocyte growth factor (HGF), and angiopoietin 1 (ANG1) different chemokines such as CXCL1, CXCL8, CXCL9, CXCL10, CCL3, and CCL4 (6) and enzymes involved in ECM remodeling (MMP9). Production and expansion of neutrophils is dependent on CSF3 (G-CSF) and its receptor CSF3R. A crucial signaling pathway for cancer inflammation is STAT3 (85), which is downstream of activated CSF3R. In response to CSF3, neutrophils upregulate the expression of BV8 (also known as prokineticin-2) that induce myeloid cell mobilization and myeloid-dependent tumor angiogenesis (86). This production of BV8 depends on the activation of STAT3 (87). The tumor angiogenesis stimulation in mice by TANs and other myeloid cells is regulated by STAT3 signaling and involves VEGFA, FGF2, and MMP9 (88). MMP9-secreting neutrophils can directly contribute in the acceleration of tumorigenesis acting on skin premalignant epithelial cells in a mouse model (89). During the early stages of carcinogenesis, TANs can mediate the initial angiogenic switch in RIP1–Tag2 transgenic mice model of pancreatic neuroendocrine tumor. The MMP9-positive neutrophils were mainly found inside angiogenic islet dysplasia as well as in tumors (90). The neutrophil depletion by GR1 or Ly6G antibodies in both transgenic and tumor transplanted mice resulted in lower levels of VEGF/VEGF receptor (VEGFR) signaling and a delay of the angiogenic switch (90). TANs lack expression of tissue inhibitors of metalloproteinases (TIMP1), rendering neutrophil-derived MMP9 more potent as angiogenesis driver in the TME than cells which produce MMP9/TIMP1 complexes (91). Neutrophils with antiangiogenic features have been reported to be able to release the endogenous angiogenesis inhibitor thrombospondin-1 in peroxisome proliferator-activated receptor (PPAR)α-deficient mice, thus preventing angiogenesis and tumor growth (92). These reports suggest that PPARα is a central transcriptional suppressor of inflammation and tumor development and could be a valuable target. Group V secreted phospholipase A2 enzymes are released by human neutrophils and enhance the proangiogenic molecules VEGFA, ANG1, and CXCL8 in an autocrine mechanism (93), but also stimulate production of the antiangiogenic isoform of VEGFA, VEGFA_165b_ (94). The functional outcome probably depends on the balance between proangiogenic and antiangiogenic factors and is still matter of investigation.

The ability of neutrophils to release several proangiogenic factors, MMPs, and other proteases (95) and to trap cancer cells *via* NET secretion (96) could promote cancer metastasis. TANs are required for the development of the premetastatic niche and metastases in murine models (97–99).

Recently, new data have brought clarity on the role of TANs and TAMs in the resistance to antiangiogenic therapy. Tumors activate PI3K signaling in all CD11b^+^ cells (both neutrophils and monocytes) (100). Inhibition of one of these cell types induces a compensatory phenomenon by the other cell types, which overcomes the angiogenic blockade. Hindering PI3K in all CD11b^+^ myeloid cells generate a long-lasting angiostatic effect (100).

## Immature Myeloid Cells (MDSC and DC)

Immature myeloid cells are innate immunity cells that infiltrate the TME, having a critical role in the proangiogenic activities and in tumor immune evasion (Figure 1). The immature myeloid cells include MDSCs and DCs, also indicated as regulatory (reg)DCs (101, 102). The immature phenotype is due to constitutive activation of STAT3 that perturbs the differentiation process of these cells. MDSCs comprise in mice and humans two distinct immature myeloid cell types: the polymorphonuclear MDSC (PMN-MDSC) characterized by neutrophil features, and the monocytic MDSC (M-MDSC) having markers of monocytes. Recently, several articles have described exhaustively both MDSC and DC phenotypic characteristics and they will not be discussed here (103–105). Several tumor-derived factors, among which CSF3, IL-1β, and IL-6, have been implicated in recruitment, activation, and expansion of MDSCs. These molecules contribute to the STAT3 activation of immature MDSCs, rendering them potent proangiogenic and immunosuppressive cells (106).

Monocytic MDSCs have been intensively studied and recognized as immunosuppressive cells as well as proangiogenic effectors in cancer (107). Murine data suggested that MDSCs are also able to differentiate into ECs (108, 109). Recent data have suggested that MDSCs in human peripheral lymphoid organs are mainly represented by PMN-MDSCs, with immunoregulatory role and are involved in the tumor-specific T cell tolerance. In the TME, there is accumulation of the M-MDSC counterpart, which is more suppressive and can rapidly differentiate to TAMs. These events might imply that targeting only one myeloid cell subset (macrophages vs. granulocytes or *vice versa*) may not be sufficient for obtaining a long-lasting immunotherapeutic effect. An investigation performed in two transplantable and two transgenic tumor murine models has shown that the tumor-induced hypoxia triggers the upregulation of CD45 tyrosine phosphatase activity in TME residing MDSCs, resulting in downregulation of STAT3 and differentiation of MDSCs into TAMs (106). There is no hypoxia in the spleens, thus CD45 downregulation of STAT3 does not occur in this organ. Use of STAT3 inhibitors in tumor-bearing mice resulted in depletion of MDSCs in the spleen but not in tumors.

Myeloid-derived suppressor cells and TAMs are regulated by metabolic constraints within the TME, and this represents a crucial factor of the signaling network regulating the expression of specific transcriptional programs with distinct protumor functions (110). Several amino acids in the TME are converted to immunomodulatory molecules such as nitric oxide, polyamines, and kinurenines. Amino acids consumption by myeloid cells decrease the availability of essential nutrients for T cells (111). The energetic metabolism of tumor-infiltrating MDCSs showed peculiar features in both mouse and human samples, such as a preferential augmented fatty acid uptake and their oxidation rather than glycolysis (112, 113). Targeting fatty acid oxidation inhibited tumor growth and combination with low dose chemotherapy blocked the MDSC immunosuppression (113). Myeloid cells in the TME produce increased fatty acid synthase in response to CSF1, which causes PPARβ/δ-dependent expression of genes, like VEGF, IL-10, and arginase 1 (Arg1), involved in the proangiogenic and immunosuppressive responses (114). A promising therapeutic approach is based on the reprogramming and the re-education of the metabolism of MDSCs in the TME, with appropriate drugs in combination with immune checkpoint inhibitors (115).

Myeloid-derived suppressor cells are also characterized by the ability to express high amounts of NADPH oxidase, which is responsible for the production of ROS in the form of superoxide anion, hydrogen peroxide, and peroxynitrite. MDSCs present also an increased expression of Arg1 and of inducible forms of nitric oxide synthase 2 genes, and they release diverse inhibitory cytokines, contributing to the immunosuppressive effects in the TME (116).

Myeloid DCs, also known as conventional (c)DCs, consists of multiple cell subsets with potent antigen-presenting cell capacity, therefore playing a fundamental role in the activation of T-cell adaptive responses against pathogens and tumor cells. However, tumor-associated cDCs or regulatory DC (regDCs) in the TME display altered functions with impaired cross-presentation capacity, express low levels of co-stimulatory molecules, and have high-proangiogenic abilities. These changes depend on diverse conditions that are established during tumor progression, for example, hypoxia, production of PGE2, IL-10, adenosine, and increased levels of lactate (117–119).

One of the major mechanisms contributing to DC dysfunction in tumor-bearing animals and in patients with different cancers is the abnormal accumulation of lipids (120). Growing evidence shows that cDCs can drive either immunosurveillance or accelerated tumor progression depending on the environment. In both mouse and human ovarian cancers, CCR6^+^ cDCs are recruited massively in the TME through the tumor-derived β-defensins and are induced to become proangiogenic cells, favoring tumor vascularization, and growth in response of tumor VEGF (121).

Depleting DC numbers in the tumor-bearing host at early stages of the disease correlates with faster tumor development in a murine model of ovarian cancer. DC inhibition at advanced stages induces on the contrary significant delays in the malignant progression (122).

During tumor progression, the hypoxia-induced regDCs remain in an immature state and acquire tolerogenic immunosuppressive properties and proangiogenic activities, for instance, by secretion of galectin-1 (123, 124). Galectin 1 is able to bind VEGFR2 and neuropilin-1, mirroring the effect of VEGF on ECs, thereby promoting angiogenesis (123–125). Moreover, regDCs are involved in the expansion and activation of Treg cells through TGFβ release, reinforcing the induction of the immunosuppressive functions of the TME (126–128). Induction of adenosine receptor A2b is triggered by the hypoxia-induced factor (HIF)-regulated elements during tumor hypoxia and is involved in skewing DCs to TH2 triggering phenotype, sustaining M2-like macrophage induction, and reinforcing tumor angiogenesis (129). Although regDCs and MDSCs have cell-type specific functional properties, their capability of regulating tumor angiogenesis in the TME appears similar to the one of M2-like TAMs and N2 neutrophils, leading to production of several soluble factors such as VEGF, FGF2, BV8, and MMP9 (130).

## Mast Cells

Mast cells (MCs) are bone marrow-derived multifunctional immune cells first identified in human tumors by Paul Ehrlich in the 1870s (69, 131). MCs and their mediators exert a host protective immune response against noxious agents, viral and microbial pathogens (132–135), but are also associated with a detrimental role in allergic diseases (69). Increased number of MCs have been observed in tumor and peritumor tissues of cancer patients (136); their role in cancer insurgence and progression is tumor dependent (69, 131). Contrasting roles of MCs in supporting or inhibiting tumor progression have been reported (131). In solid neoplasms including thyroid, gastric, pancreatic, bladder cancers, prostate adenocarcinomas, and hematological malignancies, MCs have been associated with protumorigenic activity (69, 131, 137). In breast cancer (131) and in murine model of prostatic neuroendocrine tumors (137), MCs have antitumor activities. These data clearly suggest that the role of MCs in cancer is tumor-type dependent and is tuned by the local microenvironment (Figures 1 and 2). Antitumor activities by MCs are related to their ability to induce target cell cytotoxicity by releasing TNFα or by induction of ROS. Protumorigenic activities of MCs include contribution to the induction of an acidic and immunosuppressive TME, through adenosine production in the extracellular milieu. Prometastatic functions of MCs are mediated by the release of TGFβ, which induce tumor cells to undergo epithelial to mesenchymal transition. MC releases proangiogenic factors including FGF2, VEGFA, TNFs, CXCL8 (69, 131), diverse proteases, such MMPs (MMP9 mostly), as well as chymase and tryptase that modify pro-MMPs to their active forms (5, 138). MC deficient tumor-bearing mice show a reduced angiogenesis and metastatic capacity (138, 139). In renal cell carcinoma, infiltrating MCs have been found to support angiogenesis by modulating PI3K/AKT/GSK3β/AM signaling (140). Following activation of c-KitR/SCF, MCs can release tryptase that, acting on PAR2 in tumor cells, induce endothelial and tumor cell proliferation in a paracrine manner, leading to tumor cell invasion and metastasis (141). Tryptase released by MCs sustain angiogenesis in pancreatic cancers by activating the angiopoietin-1 pathway. Tryptase producing MCs correlate with angiogenesis in locally advanced colorectal cancer patients (142). Immunohistochemical analysis showed that tryptase-positive MCs in multiple myeloma were associated with higher levels of MMP9, ANG2, and angiogenin (143) and could contribute to vasculogenic mimicry (144). Tryptase appears the key mediator for protumor activity of MCs, since it is involved in cell growth, tumor-induced angiogenesis, and invasion (145, 146), thus it appears to be a promising target for MC-related angiogenesis. Tryptase inhibitors originally designed as anti-allergic drugs could exert promising antitumor and antiangiogenic activity and could be proposed as repurposed drugs also in combination with immune therapy.

## γδT17 Cells

Gammadelta T cells are lymphoid cells characterized by unique features resembling innate cells in their capacity to recognize conserved non-peptide antigens expressed by stressed cells. They also resemble adaptive cells because of their ability to undergo clonal expansion and to develop antigen-specific memory (147). These cells are involved in the early phase of immune responses and produce pro-inflammatory factors such as IFNγ and TNFα and IL-17, activating other effector immune cells against virus, bacteria, and tumor cells but also stimulating inflammation and exacerbation of autoimmune diseases. They comprise different functional subsets.

Although there are some conflicting data on the role of γδT cells inside the TME, it is believed that the subset γδT17 cells, specialized in the IL-17 release, can actively participate in the angiogenic process (147, 148) (Figures 1 and 2). It has been shown that γδT17 cells release IL-17, CXCL8, CFS2 (also known as GM-CSF), and TNFα, and are able to support survival of MDSCs (149). Tumor cells over-expressing IL-17 showed significant tumor growth and new vessel formation (150). Since IL-17 has no direct effect on the proliferation of ECs, the proangiogenic effect is likely to be exerted through the enhancement of VEGF and/or CXCL8 by tumor cells (151). On the contrary, mice lacking IL-17 showed limited tumor growth and the vascular density in tumor tissues was decreased (152). There is evidence that IL-17 responsiveness can be an independent prognostic factor for overall survival in colorectal patients (153), high expression of IL-17 was shown to be associated with high microvessel density and was associated with VEGF production from tumor cells. More recently, it has been shown that IL-17 activates STAT3 in non-small cell lung carcinomas (NSCLC) cells and that treatment of HUVECs with IL-17 *in vitro* promoted the formation of vessel-like tubes in a dose-dependent manner (154). The GIV protein (Gα-interacting vesicle-associated protein, also known as Girdin) modulates the crucial signaling pathways in processes including macrophage chemotaxis, wound healing, and cancer metastasis and can be a target of STAT3 activation in NSCLC cell lines. IL-17-dependent STAT3/GIV signaling pathway is responsible for VEGF release from cancer cells and promotion of tumor angiogenesis, and GIV expression positively correlates with IL-17^+^ cell presence and increased microvessel densities and predicts poor survival of NSCLC patients (154).

IL-17 in the TME in the CMS-G4 fibrosarcoma tumor model was largely derived from tumor-infiltrating γδT cells, and anti-cytokine mAb treatment revealed that the γδT cells require the presence of IL-6, IL-23, and TGFβ signaling (152). In gallbladder cancer (GBC) patients, γδT17 cells are increased in peripheral blood and in the population of tumor-infiltrating lymphocytes (155). GBC patients with high γδT17, TH17, and Treg cells showed poor overall survival (155). A GBC (OCUG-1) cell line that is responsive to IL-17, treated with cell-free supernatant from γδT17 cells, upregulates VEGF production, and this effect is IL-17 dependent (155). The proangiogenic action of γδT17 cells on GBC was confirmed by protein angiogenesis array performed on cell-free supernatants derived from these cells. The assay showed IL-17-dependent upregulation of several important angiogenesis factors in OCUG-1 cells, such as VEGF, angiogenin, uPA, MMP9, CCL2, CXCL16, CSF2, and coagulation factor III, but also stimulation of production of antiangiogenic factors, including thrombospondin-1, TIMP1, serpine-1, and platelet factor 4. A recent report has shown that IL-17-secreting γδT cells are dependent on CCR6 for homing to inflamed skin (156). Drugs targeting CCR6 or factors involved in γδT17 cell proangiogenic polarization should be studied for potential use in addition with immunotherapy.

## Innate Lymphoid Cells

Innate lymphoid cells represent a recently identified heterogeneous family of mononuclear hematopoietic cells, found mostly in solid tissues (157–160). Based on their lymphoid morphology, surface antigens, transcription factor expression, and cytokine productions (TH1, TH2, and TH17-like), ILCs have been classified into three major groups, termed as ILC1, ILC2, and ILC3 (161). ILC1s are characterized by IFNγ release and are Tbet dependent; ILC2 produce type 2-cytokines, such as IL-5 and IL-13, and require GATA3 expression; ILC3s produce IL-17 and/or IL-22 and are dependent on RORγt (162). ILCs are endowed with potent pleiotropic effects in early responses against infections and are involved in several pathologies including cancer. Aberrant activation, proliferation, and functions of ILCs support severe inflammation and damages in diverse organs, including the gut, lung, liver, and skin (163–168). Whether ILCs can be defined as friends or foes in cancer insurgence and progression is still a matter of debate (157, 158, 160). ILCs are characterized by high-cell plasticity and can be easily interconverted into their different subsets upon TME stimuli [especially ILC1–ILC3 interconversion (169)].

IFNγ^+^ ILC1s have been associated with both antitumor and protumor effects (Figure 1), the latter induced by triggering of MDSCs and inducing indoleamine 2,3-dioxygenase activity (157). A protective role exerted by a novel type of ILC1-like cells has been shown in a murine model of mammary carcinogenesis (170). NK cells, that will be discussed, later have also been included in the ILC1 subclass.

ILC2s can release type 2 cytokines, such as IL-5 and IL-13, and CSF2 in response to IL-25 and IL-33. IL-13/IL-13R interaction in breast cancer and cholangiocarcinoma cells in association with recruitment and induction of TGFβ-producing MDSCs and Treg has been reported to induce tumor cell growth and migration (171), and tumor immune escape (172). Release of IL-13 by ILC2s promotes M2-like TAM polarization and amplification (172).

Among the ILC subgroups, ILC3s are the more investigated for their contribution to carcinogenesis. They comprise several subsets: lymphoid tissue inducer (LTi) cells, first discovered for their function in the formation of lymphoid tissue during organogenesis, NCR (NKp46, NKp44)^+^ ILC3 and NCR^−^ ILC3. Overall, the pro-tumor activities of ILC3s are mainly linked to the induction of chronic inflammation by secretion of IL-17 and IL-22, in particular in the gut, through their response to IL-23 (173).

ILC3s preserve epithelial integrity and maintain tissue homeostasis by secretion of IL-22. Production of IL-17 by ILC3s can have a role in promoting tumorigenesis, tumor growth, and angiogenesis (174–176). Growing evidence from mouse tumor models marks ILC3s as cells involved in the recruitment of MDSCs, Treg cells, and in the promotion of M2-like macrophages in the TME. At the moment, the real contribution in human cancers remains to be fully elucidated (177, 178). ILC3s have also been shown to play a role in carcinogenesis in models of bacteria-induced colorectal cancer, through the release of IL-22 (179). The involvement of LTi-like ILC3s has been shown in the induction of tumor migration *via* lymphatics in patients with triple-negative breast cancers (180). In the 4T1.2 syngeneic mouse breast model, ILC3s are recruited in the primary tumor through CCL21, and then they trigger tumor stromal cells to release CXCL13, which leads to the induction of lymphotoxin and receptor activator of nuclear factor *κ*-B ligand, that in turn promotes lymphangiogenesis and stimulate tumor cell motility (180). A correlation exists between invasive aggressive behavior in breast cancer patients and gene expressed by ILC3s such as CXCL13, CCL19, CCL21, and CXCR5 and CCR7 (181). ILC3s have been shown to promote the formation of tertiary lymphoid structures (TLS), involved in tumor progression and lymph nodal metastasis (182). The protumor or antitumor roles of TLS are still debated (183, 184). NKp46^+^ NKp44^+^ LTi-like ILC3s are present in the TME near intra-tumor TLS and may interact directly with tumor cells by sensing and recognizing transformed cells through the NKp44 receptor. Tumor-infiltrating NKp46^+^ NKp44^+^ LTi-like ILC3s are endowed with ability to release several types of pro-inflammatory cytokines and chemokines, and their increased numbers correlated with intra-tumor TLS and predict favorable clinical outcome (185). Accumulation of neuropilin (NRP)1^+^ LTi-like ILC3s has been found in inflamed tissues of patients with chronic obstructive pulmonary disease and in smokers, in association with VEGF production (186). Immunohistochemistry analysis of inflamed tissues revealed that the majority of RORγτ^+^NRP1^+^ cells were co-localized with blood vessels and in the alveolar parenchyma, suggesting their contribution to angiogenesis and induction of lung TLS. Apart from IL-22 and IL17, the pro-inflammatory LTi-like NRP1^+^ ILC3 subset was also found to release CSF2, TNFα, B-cell-activating factor, and CXCL8, possibly contributing to angiogenesis.

Due to the recent discovery of the non-NK ILCs and the incomplete knowledge of the role in tumor and angiogenesis, targeting strategies have not been yet developed.

## NK Cells

Natural killer cells are bone marrow-derived large granular effector lymphocytes of the innate immune system that can potentially control tumor growth by their cytotoxic activity (187), which are now classified as a subset of ILC1 (161). Based on surface density expression of CD56, an isoform of the human neural cell adhesion molecule, and of CD16, the low-affinity Fcγ receptor, two main subpopulations of peripheral blood NK cells have been identified in humans: the CD56^dim^CD16^+^ and the CD56^bright^CD16^−/low^ NK cell subset, representing about 90–95% of peripheral blood NK cells and about 5–10% of peripheral blood NK cells, respectively. CD56^dim^CD16^+^ NKs can release high quantity of perforin and granzymes and are cytotoxic when encountering cells with high-activating ligands and low inhibitory (mostly class I MHC) ligands or when mediating antibody-dependent cell cytotoxicity (187). Although weak long-term cytokine producers, these cells have the ability to quickly (2–4 h) secrete high amounts of cytokines (188, 189). CD56^bright^CD16^−/low^ NKs, are poorly cytotoxic, but can release several cytokines, including IFNγ, TNFα, and GM-CSF. However, there is an increasing awareness of the complexity of NK cell subsets and the role of the TME (190–193). Mature NK cells express the PD-1 receptor, and engagement with the programmed death-ligand 1 (PD-L1) ligand results in impaired antitumor NK cell activity (194, 195). Disruption of this PD-1/PD-L1 by blocking antibodies partially restores their antitumor activity (194, 195). Another recently identified NK checkpoint is the IL-1R8 (also known as SIGIRR, or TIR8), which is expressed on human and murine NK cells (196). Mice lacking IL-1R8 are protected against chemically-induced tumors and metastatic dissemination (196). Mice lacking the cytokine-induced SH2-containing protein CIS also had protection toward chemically induced tumors and metastatic disease (197).

A third NK cell subset has been identified in the decidua during pregnancy, termed decidual or uterine NK cells (dNK). dNK cells acquire the CD56^superbright^CD16^−^KIR^+^ phenotype (198), are poorly cytotoxic, and secrete proangiogenic cytokines, including VEGF, placental growth factor (PlGF), CXCL8, and IL-10 (198–200) and are critical for decidual vascularization and spiral artery formation (199, 201). Early on in pregnancy, dNK increase up to 70% of the local lymphocytes and 30–40% of all decidual cells (202). While it has been exhaustively demonstrated that NK cells have important proangiogenic roles in the uterine vasculature, their contribution to tumor angiogenesis still represent a poorly explored topic (Figure 1). The TME has been extensively reported to be crucial in shaping NK cell functions (203). We were the first to report a proangiogenic NK cell polarization in peripheral blood (TANKs) and tumor-infiltrating NK cells (TINKs) (204) in NSCLC patients. We showed that the CD56^bright^CD16^−^ NK cells, the predominant subset infiltrating NSCLC tissues and a minor subset in adjacent lung and peripheral blood, are associated with VEGF, PlGF, and IL-8 production (Figure 2). Functional assays indicated that supernatants derived from NSCLC CD56^bright^CD16^−^ NK cells induce EC chemotaxis and formation of capillary-like structures *in vitro*, and that these effects were even stronger in TANKs isolated from subset of squamous carcinoma patients than in adenocarcinoma.

TGFβ is associated with dNK polarization (205, 206) and is present in the TME. A combination of TGFβ, hypoxia, and a demethylating agent induces a dNK-like phenotype in healthy donor NK cells (207). A recent report indicated that TGFβ converted NK cells into other ILC1 subpopulations that were unable to control local tumor growth and metastasis (208). We observed that TGFβ1 upregulates VEGF and PlGF in healthy donor NK cells (204).

Tumor-infiltrating NK cells operate within a hypoxic TME. Hypoxia has been extensively reported to modulate immune cell response as well as driving angiogenesis (209). Murine NK cells genetically depleted of HIF1α continued to have impaired cell cytotoxicity, yet tumors grew more slowly in these mice (210). Tumors in these mice had numerous immature vessels with hemorrhages that resulted in severe hypoxia, which favored metastasis. Genetic inactivation of STAT5, which is necessary for NK cell-mediated cancer immunosurveillance, increases VEGFA in NK cells and stimulates angiogenesis in mouse lymphoma models and on healthy donor-derived NK cells (211). The aminobiphosphonate zoledronic acid, largely employed as an immunomodulatory agent and able to decrease VEGF levels, has been surprisingly found to synergize with IL-2 in inducing proangiogenic features in TINKs, acting on VEGF/VEGFR1 axis (212). Thus, therapeutic intervention could act as a double edge sword in NK cell response to tumors.

## Pharmacological and Immunotherapeutic Combination Targeting the TME

Extensive studies on TME led to a shift from a tumor-centered view of cancer onset to the role of a more complex tumor ecosystem in which cellular and molecular components are as influential as cancer cells themselves for cancer development and metastatic behavior. This knowledge led to the rapid development of therapeutic approaches aimed at restoring altered/aberrant host immune cell response, by accelerating/pushing efficient tumor eradication, stimulating immune cells of the host (213). The use of immune checkpoint blockers (ICBs) induces reactivation of key immune cell players and has been demonstrated to have great clinical benefits in several tumors (214). Available ICBs target cytotoxic T lymphocyte-associated protein 4 (CTLA-4), programmed cell death 1 (PD-1) receptor, and its ligand PD-L1. Known ICBs are: Ipilimumab, a mAb-blocking CTLA4, approved in patients with unresectable or metastatic melanoma. Pembrolizumab, a mAb-blocking PD-1, initially licensed for use in patients with unresectable or metastatic melanoma experiencing disease progression on ipilimumab. Pembrolizumab has been recently made available for other types of cancer (metastatic Non-Small Cell Lung Cancer, Head and Neck Cancer, Hodgkin’s Lymphoma, Urothelial Carcinoma and Gastric Cancer). Nivolumab is another mAb directed to PD-1 approved for use in individuals with unresectable or metastatic melanoma non-responding to other treatments, as well as in patients with metastatic NSCLC, or after platinum-based chemotherapy. Atezolizumab is a PD-L1-blocking antibody for the treatment of locally advanced or metastatic urothelial carcinoma. Despite the strong clinical success of cancer immunotherapy with checkpoint inhibitors and other immune modulating agents, most patients still do not experience a durable response (215) and many do not respond at all. To overcome this issue, several strategies combining immune to targeted therapy have been developed.

The gut microbiome, which has a significant influence on the local and systemic immune system, can influence the outcome of ICB therapy in preclinical mouse models and humans (216–219). A recent study on the gut and oral microbiome of a cohort of melanoma patients undergoing an anti-PD-1 therapy revealed crucial differences in the diversity and composition of the patients’ gut microbiome of responders vs. non-responders (216). Analysis of patient fecal microbiome in responding melanoma patients indicated significantly higher relative abundance of bacteria of the *Ruminococcaceae* family that also correlated with presence of CD8^+^ T cells in the TME. Fecal microbiota transplantation in germ-free recipients showed that mice which had been transplanted with stool from responders to anti-PD-1 therapy had significantly reduced tumor size and higher density of CD8^+^ T cells in comparison to mice receiving stool from non-responders to PD-1 blockade (216). Another recent study on different epithelial tumors in mice and patients indicated correlations between clinical responses to ICBs and the relative abundance of *Akkermansia muciniphila* (217). Hence, the gut microbiome can strongly influence the outcome of cancer patients receiving PD-1 blockade therapy. However, the mechanisms related to these immunomodulatory effects of *A. muciniphila* remain elusive. It is conceivable that an integral intestinal barrier is associated with a minor systemic inflammation, and specific bacterial families such as *Ruminococcaceae* and/or *A. muciniphila* may induce beneficial bacterial metabolites that prevent leaky colon and systemic immunosuppression, paving the way to the possibility to manipulate the gut ecosystem to implement ICB therapy (218).

**Figure 3:**
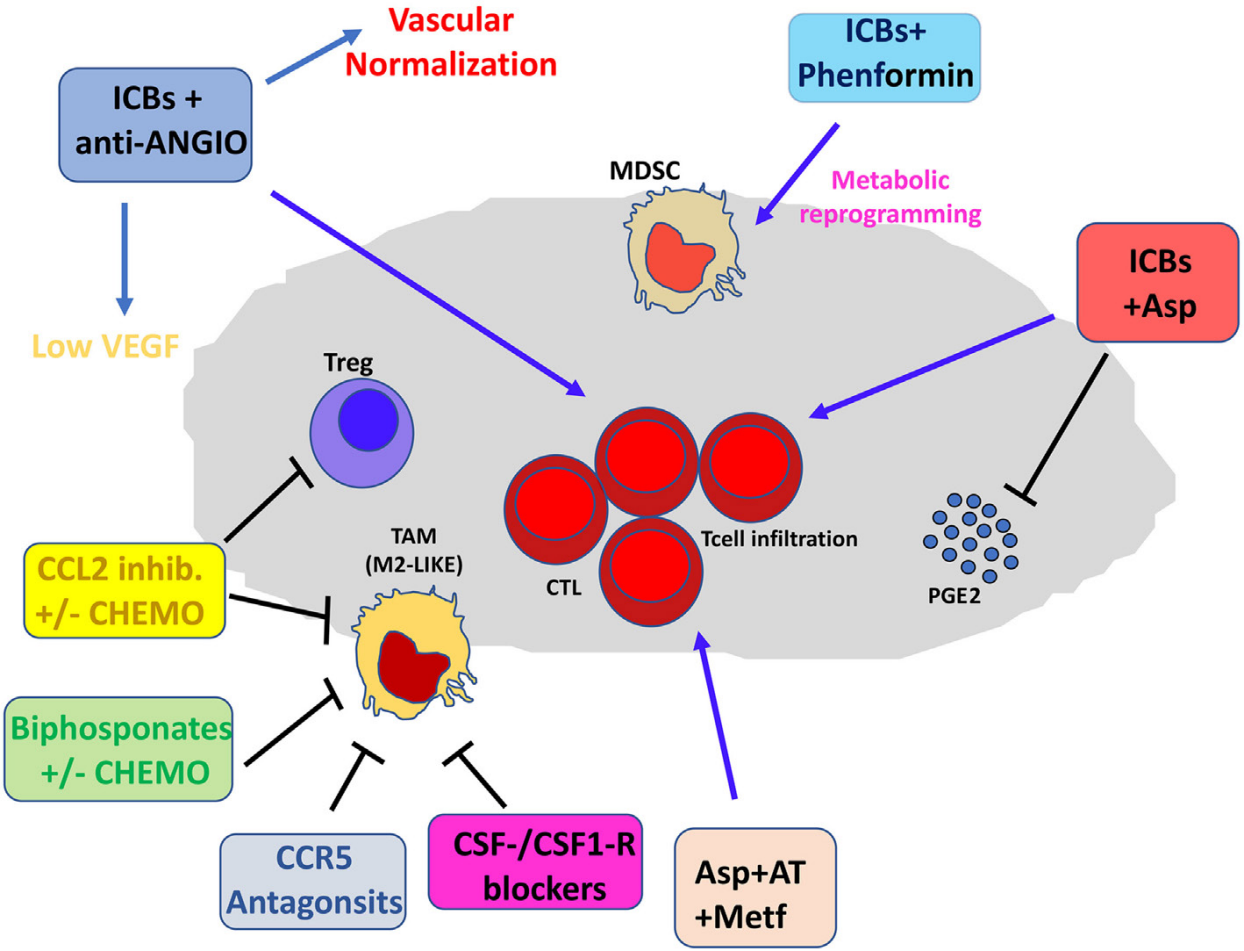
Pharmacological and immunotherapeutic combination targeting the tumor microenvironment (TME). Immune checkpoint blockers (ICBs) can be employed in diverse pharmacological combinations resulting in clinical benefit for patients. ICBs + antiangiogenic agents result both in inhibition of aberrant angiogenesis and vascular normalization with subsequent efficient T cell infiltration. CCL2 inhibitors combined with chemotherapy dampen induction of M2-like tumor-associated macrophages (TAMs) in the TME and T regulatory (Treg) proliferation. Bisphosphonates + chemotherapy target M2-like TAMs. Anti-Angio, VEGF inhibition and eventually angiopoietin-2 blockade; Asp, aspirin; AT, atenolol; Metf, metformin; Chemo, standard chemotherapeutic drugs.

**Figure 4:**
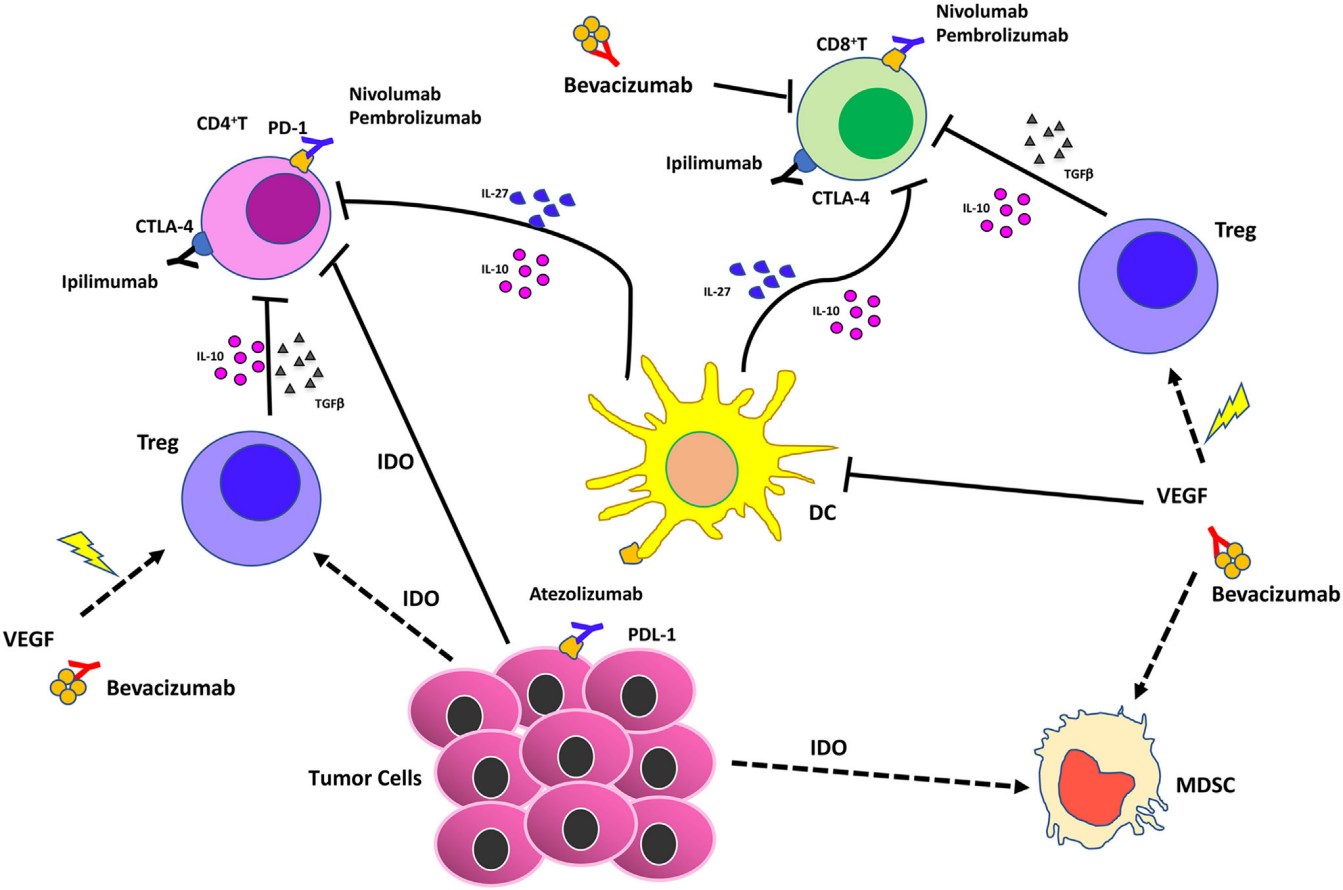
Effects of vascular endothelial growth factor (VEGF) inhibition combined with immunotherapy in the tumor microenvironment. Immune checkpoint blockers (ICBs) combined with antiangiogenic drugs act synergistically on different cell of innate immunity by (i) reducing VEGF in the tumors that supports angiogenesis; (ii) supporting vascular normalization to stabilize blood vessels and enhance therapeutic agent delivery, T cell infiltration, and activation; (iii) blocking dendritic cells, myeloid-derived suppressor cells, T regulatory (Treg)-mediated immunosuppression.

All recent preclinical and clinical data suggest that the localization, quality, and quantity of non-cancerous cells, including lymphoid and myeloid cells, within the TME play a major role in shaping response to immune checkpoint blockade (Figures 3 and 4). Other TME cells, such as fibroblast and ECs, could contribute to shaping the immune contest. An emerging role is demonstrated for the angiogenic factor VEGF.

Vascular endothelial growth factor blocks T cell infiltration into the tumor by inhibition of adhesion molecules on ECs (220). VEGF has also been reported to inhibit antigen presentation by DCs, to enhance the Treg expansion, and to mediate PD-1 upregulation on tumor-infiltrated T cells (221, 222). Antiangiogenic treatments such as anti-VEGF antibody bevacizumab and the diverse multi-tyrosine kinase receptor inhibitors targeting the VEGFR family have been largely employed in the clinic, combined with chemotherapy, in particular in colorectal and renal cancer. They have shown significant but moderate benefits in patients’ overall survival (223). Excessive pruning of vessels following anti-VEGF treatment has been reported to associate with increased hypoxia that, through upregulation of CXCL12/CXCR4 axis and HIF1α, supports M2-like TAM, MDSC, and Treg recruitment, thus supporting tumor progression (223). Tumors show considerable variation in their responses to antiangiogenic therapy, however, given the immunosuppressive action of VEGF (47, 222, 224), VEGF inhibitors could combine with the ICBs to enhance therapeutic effects.

Therefore, combination with antiangiogenic agents, and/or anti-inflammatory drugs has a strong rationale (47, 225, 226) but it is still in its infancy. Preclinical and clinical studies in renal cancer showed that the combination of anti-CTLA-4 with sunitinib (227) resulted in decreased Treg and increased CD8^+^ T cell infiltration (Figure 4). Conversely, increased PD-L1 expression has been observed following treatments with sorafenib, sunitinib, or bevacizumab in a HIF1α-dependent and -independent manner (228). Growing evidence supports the notion that the targeting of VEGF signaling could result in the induction of tumor vasculature normalization, enhancement of immune cells extravasation, and synergy with immunotherapy (229–231). The combination of bevacizumab and ipilimumab has been reported to be associated with clinical benefits in patients with melanoma (232), and has been found to target Galectin-1 (233–235). Blocking of VEGFA and angiopoietin-2 using a bispecific antibody in murine models resulted in activation of cytotoxic T lymphocytes, which upregulated PD-L1, and inhibition of PD-1 axis further improved the efficacy of this therapy (236). Another rationale for the combination of ICBs and antiangiogenic agents is that antiangiogenic agents “normalize” the tumor vasculature, inducing intra-tumor high endothelial venules, thus favoring enhanced T-cell infiltration, antitumor CTL activity, and tumor cell destruction (236, 237). ICBs in combination with antiangiogenic agents may act as a promising strategy also to dampen the proangiogenic features of immune-infiltrating cells, such as TAMs, MDSCs, and NK cells, acting as re-polarizing agents (226, 238, 239).

Chronic inflammation, another relevant hallmark of cancer (3), directly stimulates angiogenesis to support tumor progression (5, 7) and immune suppression (16, 17, 107, 225, 226). The immunosuppressive inflammatory TME is a key obstacle to cancer immunotherapy (Figures 3 and 4). Thus, targeting chronic inflammation could be one strategy to combat the immunosuppressive TME and enhance the activities of ICBs. One example is targeting the PI3Kγ, which has a strong effect on myeloid cells, preventing immune suppression and enhancing the effects of ICBs *in vivo* (240, 241) (Figures 3 and 4). Another example of therapy that could synergize with ICBs is targeting the CXCR2 axis, which recruits neutrophils into the premetastatic niche (98).

**Figure 5:**
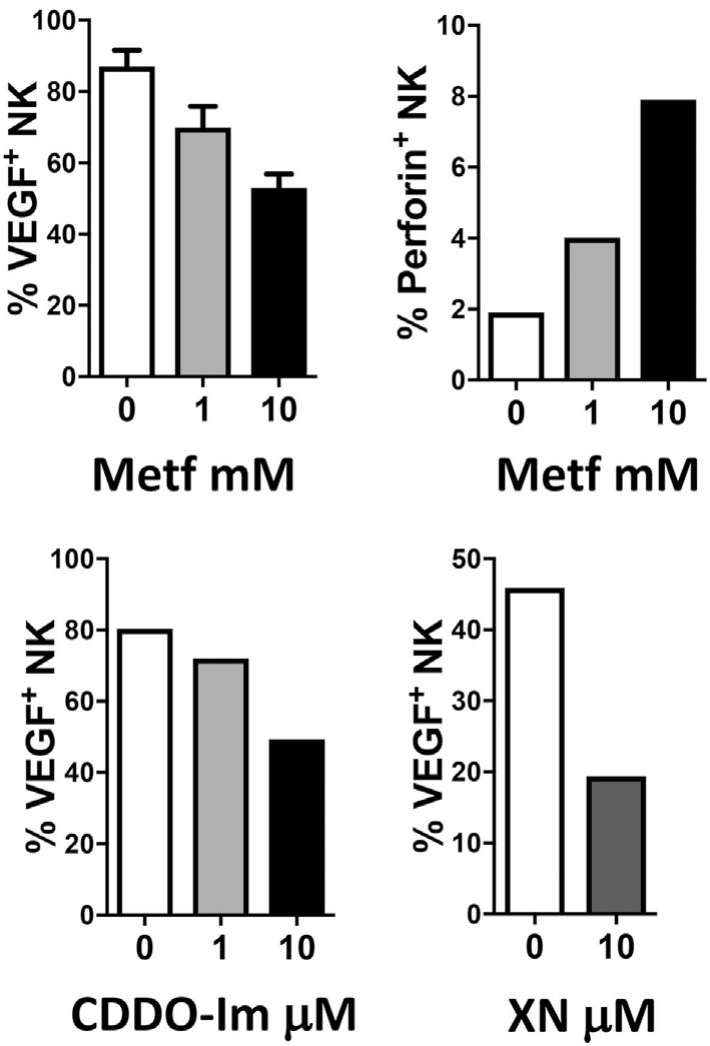
Examples of effects of repurposed drugs and phytochemicals on natural killer (NK) cell repolarization. The biguanide metformin (Metf), the synthetic triterpenoid 1[2-Cyano-3,12-dioxooleana-1,9(11)-dien-28-oyl]imidazole (CDDO-Im) and the hop flavonoid xanthohumol (XN) can decrease vascular endothelial growth factor (VEGF) production in non-small cell lung cancer associated NK cells and at the same time can upregulate perforin production. Graphs show data obtained from multicolor flow cytometry analysis of total NK cells (CD45^+^CD14^−^CD3^−^CD56^+^ cells) from peripheral blood samples of patients with non-small cell lung carcinomas (as in 204, protocol number 0024138/2013), exposed for 24 h to the compounds at indicated concentrations. These examples sustain the action of anti-inflammatory chemopreventive drugs in innate immune cells repolarization supporting the rationale for future combinations with immunotherapy.

The combination of anti-inflammatory agents with ICBs can be exploited to support immunotherapy. Regular use of aspirin, the most commonly employed nonsteroidal anti-inflammatory drug, has been widely reported to reduce incidence and mortality of colorectal cancer (242) and many other adenocarcinomas (243). A recent U.S. population-based study reported a stronger survival association of post-diagnosis aspirin use in CRC patients with lower-level PD-L1 expression when compared with those with higher-level of PD-L1 expression (244). Experimental data supported a synergistic effect between aspirin and anti-PD1 antibody in mutant Braf(V600E) melanoma cells (245). The synergistic effects resulted also in increased T cell-mediated immune responses and decreased PGE2 production (245). In experimental models, we showed that aspirin or the beta-blocker agent atenolol can augment the activity of metformin, a biguanide largely employed in type 2 diabetes management and that have been associated to reduced risk of developing diverse cancers, including breast cancers (Figure 4), targeting both neoplastic cells and the TME (246, 247). Metformin and phenformin affect the angiogenesis pathway (248–250) and modulate the immune response and the microbiome (251, 252). Phenformin enhances PD-1 immunotherapy (115). CDDO-Im (a synthetic triterpenoid: 1[2-Cyano-3,12-dioxooleana-1,9(11)-dien-28-oyl]imidazole) has an extensive documentation as an immunomodulation agent (253–255), and xanthohumol (XN) (a prenylated chalcone flavonoid) is an antileukemia agent (256–259) and is a polarizing agent in murine models of breast cancer (260). Phytocompounds and their synthetic derivatives are able to polarize macrophages inducing anti-tumorigenic phenotype/functions (253, 260–262). For example, we show that NSCLC patient TANKs treated with metformin, CDDO-Im, and XN decreases VEGF production (Figure 5) and increases perforin content. Thus, we would like to indicate the use of non-toxic or low-toxic re-polarization agents endowed with anti-inflammatory chemopreventive properties to be combined with ICBs.

## Conclusion

The immune checkpoint inhibitors have posed a distinct milestone in cancer therapy. However, several patients do not respond to the ICBs, or have a relapse, with eventual long-term toxicity (i.e., autoimmune diseases). The polarized TME is crucial in the outcome of the patient response to an ICB, thus treating an inflamed or vascularized TME, could theoretically enhance the efficacy of these drugs. We suggest to combine ICBs with drugs that inhibit VEGF (232) or to employ drugs that eliminate the protumor inflammatory cells (for example, trabectedin to eliminate TAMs) or to treat with anti-inflammatory agents that will “re-polarize” the immune cells, for example, the repurposed drugs (metformin) and phytochemicals and their synthetic derivatives (CDDO-Im and XN) or both. Since phytochemicals and their synthetic derivatives often protect the cardiovascular system from chemotherapy induced damage (248, 263, 264), we propose, as a first-line therapy for difficult and metastatic tumors, to pretreat with phytochemicals or synthetic derivatives, then continue treatment and add sequentially a VEGF blocker, ICBs, and chemotherapy (to trigger the immunogenic cell death). This will set the stage for the ICBs to become highly effective in additional patients.

## Author Contributions

All authors listed have made a substantial, direct, and intellectual contribution to the work and approved it for publication.

## Conflict of Interest Statement

The authors declare that the research was conducted in the absence of any commercial or financial relationships that could be construed as a potential conflict of interest.
